# Reciprocal deregulation of NKX3.1 and AURKA axis in castration-resistant prostate cancer and NEPC models

**DOI:** 10.1186/s12929-021-00765-z

**Published:** 2021-10-08

**Authors:** Moloud Aflaki Sooreshjani, Mohini Kamra, Amina Zoubeidi, Kavita Shah

**Affiliations:** 1 Department of Chemistry and Purdue University Center for Cancer Research, 560 Oval Drive, West Lafayette, IN 47907 USA; 2 Urologic Sciences, University of British Columbia, Vancouver, V6H 3Z6 Canada

**Keywords:** AURKA, NKX3.1, Castration-resistant prostate cancer, Prostate cancer

## Abstract

**Background:**

NKX3.1, a prostate-specific tumor suppressor, is either genomically lost or its protein levels are severely downregulated, which are invariably associated with poor prognosis in prostate cancer (PCa). Nevertheless, a clear disconnect exists between its mRNA and protein levels, indicating that its post-translational regulation may be critical in maintaining its protein levels. Similarly, AURKA is vastly overexpressed in all stages of prostate cancer (PCa), including castration-resistant PCa (CRPC) and neuroendocrine PCa (NEPC), although its transcripts are only increased in ~ 15% of cases, hinting at additional mechanisms of deregulation. Thus, identifying the upstream regulators that control AURKA and NKX3.1’s levels and/or their downstream effectors offer an alternative route to inhibit AURKA and upregulate NKX3.1 in highly fatal CRPC and NEPC. AURKA and NKX3.1 have not linked to each other in any study to date.

**Methods:**

A chemical genetic screen revealed NKX3.1 as a direct target of AURKA. AURKA-NKX3.1 cross-talk was analyzed using several biochemical techniques in CRPC and NEPC cells.

**Results:**

We uncovered a reciprocal loop between AURKA and NKX3.1 in CRPC and NEPC cells. We observed that AURKA-mediated NKX3.1 downregulation is a major mechanism that drives CRPC pathogenesis and NEPC differentiation. AURKA phosphorylates NKX3.1 at three sites, which degrades it, but AURKA does not regulate NKX3.1 mRNA levels. NKX3.1 degradation drives highly aggressive oncogenic phenotypes in cells. NKX3.1 also degrades AURKA in a feedback loop. NKX3.1-AURKA loop thus upregulates AKT, ARv7 and Androgen Receptor (AR)-signaling in tandem promoting highly malignant phenotypes. Just as importantly, we observed that NKX3.1 overexpression fully abolished synaptophysin and enolase expression in NEPC cells, uncovering a strong negative relationship between NKX3.1 and neuroendocrine phenotypes, which was further confirmed be measuring neurite outgrowth. While WT-NKX3.1 inhibited neuronal differentiation, 3A-NKX3.1 expression obliterated it.

**Conclusions:**

NKX3.1 loss could be a major mechanism causing AURKA upregulation in CRPC and NEPC and vice versa. NKX3.1 genomic loss requires gene therapy, nonetheless, targeting AURKA provides a powerful tool to maintain NKX3.1 levels. Conversely, when NKX3.1 upregulation strategy using small molecules comes to fruition, AURKA inhibition should work synergistically due to the reciprocal loop in these highly aggressive incurable diseases.

**Supplementary Information:**

The online version contains supplementary material available at 10.1186/s12929-021-00765-z.

## Background

Aurora kinase A (AURKA), a serine/threonine kinase, is essential for mitosis in normal cells. AURKA is overexpressed in numerous solid and hematological cancers. AURKA protein is increased in 100% of prostate intraepithelial neoplasia (PIN) lesions, and in vast majority of prostate tumors (> 94%) [1]. In contrast, AURKA transcripts were upregulated in only 15.4% of prostate cancer (PCa) and 76.3% of BPH specimens [2], indicating that post-translational stabilization of AURKA is a critical factor in promoting its deregulation. AURKA levels were also significantly higher in local and metastatic CRPC tumor specimens as compared to hormone-naïve PCa samples [3]. AURKA overexpression is also a hallmark of de novo and treatment-induced neuroendocrine prostate cancer (NEPC) [4]. Many AURKA-selective inhibitors are in clinical trials. Alisertib (aka MLN8237), one of the selective-AURKA inhibitors, is currently being used in many Phase I and II trials against various cancers, including in CRPC [5]. Nevertheless, no AURKA-targeted drug has been approved yet, partly because alisertib has shown efficacy in only ~ 20–25% patients at the best, particularly in solid cancers. Furthermore, as AURKA is an essential kinase, its inhibition may cause substantial collateral toxicity in normal tissues. In contrast, AURKA inhibition in combination with chemotherapy, radiation, HDAC or MYCN inhibitors improves the efficacy in up to 40–50% patients [6–8]. Thus, an alternate approach is to identify the upstream regulators and downstream targets of AURKA, which could potentially be used as therapeutic intervention points to target AURKA-induced malignancy either alone or in combination with AURKA inhibitors. Previously, we have identified a few such oncogenic downstream targets and upstream regulators, which both regulate and are regulated by AURKA in a feedback loop [9–14]. Thus, specific inhibition of these substrates provides an effective alternate approach to indirectly modulate AURKA with potentially much less toxicity.

The present study focuses on one such feedback loop, which was discovered between AURKA and a tumor-suppressor NKX3.1 in CRPC and NEPC cells. We have identified NKX3.1 as a direct substrate of AURKA by employing a pioneering global screen [9, 15–18]. Unlike AURKA, which is ubiquitously expressed, NKX3.1 is a prostate-specific transcription factor, which is essential for the development and maintenance of prostate and testes [19]. NKX3.1 is also a tumor suppressor gene, situated on chromosome 8p21.2, which shows loss of heterozygosity (LOH) in up to 89% of high-grade prostatic intraepithelial neoplasia (HG-PIN) and up to 86% of prostatic tumors [20]. NKX3.1 is fully lost in up to 78% of metastatic lesions and 34% of CRPC [21]. Targeted disruption of NKX3.1 in mice causes prostatic epithelial hyperplasia and PIN [22]. Moreover, when combined with Pten disruption, loss of one or both NKX3.1 alleles causes more aggressive and rapid PCa [23]. Importantly, loss-of-function of Nkx3.1 and Pten facilitates androgen independence following castration [24]. Subsequently, the authors reported that Nkx3.1-Pten mice acquire androgen-independence even before the manifestation of PIN or PCa [25]. These findings indicate that loss of NKX3.1 is intimately linked with CRPC progression.

While the tumor-suppressive functions and loss of NKX3.1 are well established, a contradiction exists between its mRNA and protein levels in PCa. Most of the studies reported that mRNA levels of NKX3.1 are either increased or unchanged in PCa tissues compared to normal tissues [26]. In contrast, NKX3.1 protein was uniformly downregulated in IHC studies [22, 27]. These findings suggest that NKX3.1 downregulation at the post-translational stage may contribute significantly to PCa pathogenesis, which prompted us to examine whether the reciprocal levels of AURKA and NKX3.1 in PCa are related to each other.

## Methods

### Cell lines and antibodies

C4-2, 22Rv1, HEK-293T and Phoenix cells were purchased from American Type Culture Collection (ATCC; Manassas, VA, USA) and maintained according to the manufacturer’s instructions. 49F cells were obtained from Dr. Amina Zoubeidi and maintained in RPMI medium, 10% FBS along with 10 μM enzalutamide (MedChemExpress, NJ, USA). The details of the antibodies used in this study are included in Additional file 1: Table S1.

### AURKA and NKX3.1 shRNAs

Cloning of human AURKA and NKX3.1 shRNAs have been reported before [9, 28]. Lentiviruses were generated as before [29].

### Plasmids, expression and purification of AURKA and NKX3.1

Cloning of AURKA and NKX3.1 were reported before [9, 28]. Kinase inactive (D274N)AURKA was generated using site directed mutagenesis. NKX3.1 mutants were created by site-directed mutagenesis. AURKA kinase was expressed and isolated from SF9 cells [9]. AURKA and NKX3.1 retrovirus were generated as before [30].

### In vitro phosphorylation assays

6x-His-tagged AURKA was purified using Ni-NTA beads. 6x-His-TPX2 was expressed and isolated from *Escherichia coli*. To remove the background signal, the AURKA-TPX2 complex was treated with kinase buffer (50 mM Tris, 10 mM MgCl_2_) containing ATP (100 μM) for 1 h at 30 °C. Subsequently, the beads were washed three times with kinase buffer, followed by the addition of ~ 2 μg of 6x-His-tagged WT type or mutant NKX3.1 and 2 μCi of [γ-^32^P] ATP for 25 min. The reaction mixture was boiled in SDS-PAGE dye for 5 min, proteins were separated using SDS-PAGE and exposed to X-Ray film.

### Immunofluorescence

Immunofluorescence was performed as before [31]. PCa cells were plated on poly-lysine-coated coverslips for 16 h. The cells were treated with respective lentivirus (30 h) or Alisertib (1 μM for 12 h). The cells were fixed, permeabilized and blocked with PBS/0.1% triton X-100/2% BSA solution. The coverslips were incubated with substrate-specific antibodies overnight at 4 °C, followed by dye-conjugated secondary antibody for 2 h in dark. Cells were counterstained with DAPI (Sigma, MO, USA) for 5–10 min (dilution of 1:50,000). Images were taken using a Nikon Eclipse E600 microscope (Nikon Instruments, Melville, NY).

### Real-time qPCR

Real-time qPCR experiments were conducted as reported before [32]. The primers are listed in Additional file 1: Table S2. Each experiment was carried out in triplicate at least three independent times.

### Cycloheximide assay

The cells were seeded in 6-well plates for 12 h prior to infection. Subsequently, corresponding retro- or lentiviruses were added for an additional 32 h. Cycloheximide (20 μg/ml) was then added for the times indicated in the figures prior to lysis. The cell lysates were subjected to Western blot analysis.

### Ubiquitylation assay

Ubiquitylation assay was performed as described before [14]. Briefly, C4-2 and 22Rv1 cells were infected with 6x-His-Ubiquitin retrovirus along with either AURKA or NKX3.1 (WT or mutant) retrovirus for 30 h, followed by MG132 (10 μM) addition for 12 h. The corresponding lysates were incubated with either  Ni-NTA beads or specific antibodies for 4 h. The proteins were separated by SDS-PAGE, transferred on PVDF membrane and the ubiquitylated proteins were detected using either 6x-His or substrate-specific antibody.

### Isolation of cytosolic and nuclear fractions

C4-2 and 22Rv1 cells were washed twice with chilled PBS, resuspended in buffer A (10 mM Tris pH 7.9, 10 mM KCl, 0.5 mM DTT, 0.05% NP40, 1.5 mM MgCl_2_, and 1 mM PMSF) and placed on ice for 10 min, followed by centrifugation at 3000 rpm at 4 °C (10 min). To separate the nuclear fraction, the pellet was resuspended in buffer B (300 mM NaCl, 5 mM Tris pH 7.9, 1.5 mM MgCl_2_, 0.2 mM EDTA, 0.5 mM DTT, 26% glycerol (v/v) and 1 mM PMSF). The suspension was homogenized using a 27½ gauge needle (ten times). The lysates were placed on ice for 30 min and the nuclear fraction was separated by centrifugation at 24,000×*g* at 4 °C for 20 min. The cytosolic and nuclear extracts were further analyzed by Western blotting [33].

### Chemotaxis assay

Migration assay was performed using Boyden chambers as reported previously [18].

### MTT assay

The MTT assay was conducted as before [34].

### Clonogenic assay

Clonogenic assay was conducted as performed earlier [18].

### Neurite outgrowth assay

49F NEPC cells were seeded in a 6-well plate at a density of 5 × 10^4^ cells/well. After 12 h, the cells were infected with the respective retroviruses to initiate ectopic overexpression of the wild-type and phospho-resistant NKX3.1. 36 h post infection, the cells were washed with PBS and imaged under AmScope light microscope. The definition of a neurite points to “an extension from the cell body equivalent or greater than 1× the cell body width” [35]. Bright field images were imported in ImageJ software and neurite length was calculated as fraction of cell body width. This length was normalized against the vector-treated cells. Ten different fields of cells were used for quantification from five different replicates.

### Statistical analysis

All data are displayed as mean ± SEM of three or more experiments. Statistical analysis was performed using GraphPad Prism (version 6.07). Statistical significance of difference was determined by the one-way analysis of variance (ANOVA) followed by Bonferroni’s post hoc test. P < 0.05 was considered statistically significant.

## Results

### AURKA directly phosphorylates NKX3.1 in vitro

**Fig. 1:**
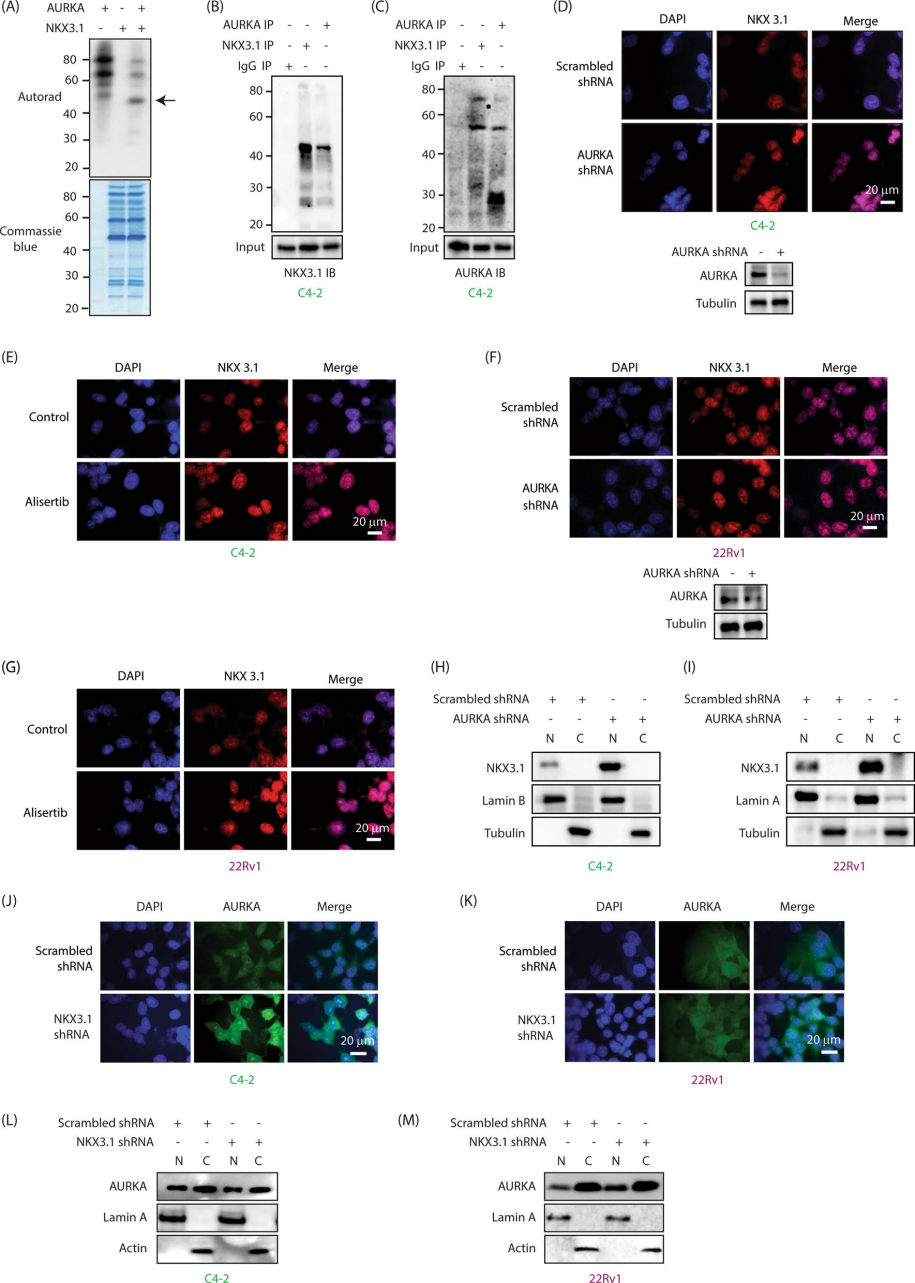
AURKA associates with NKX3.1 and phosphorylates it. **A** AURKA phosphorylates NKX3.1 in vitro. Recombinant NKX3.1 was incubated with 6-His-AURKA-TPX2 complex for 30 min. The proteins were separated by SDS-PAGE and visualized by autoradiography. The top panel is the autoradiograph, while the lower panel is the corresponding Coomassie blue-stained gel. All assays were repeated at least three times. **B** NKX3.1 and AURKA bind each other in C4-2 cells. AURKA was immunoprecipitated and its association with NKX3.1 analyzed. IgG was used as the negative control, and NKX3.1 IP was used as a positive control. **C** NKX3.1 and AURKA bind each other in C4-2 cells. NKX3.1 was immunoprecipitated and its binding with AURKA was analyzed. IgG was used as the negative control, and AURKA IP was used as a positive control. **D** AURKA knockdown does not impact the subcellular localization of NKX3.1 in C4-2 cells. Immunofluorescence micrographs of C4-2 cells infected with either scrambled or AURKA shRNA followed by probing with NKX3.1 antibody (red). Nuclear counterstain is represented by DAPI (blue). (Scale bar = 20 μm). AURKA knockdown was confirmed using Western blot analysis. Images for control cells (having much lower NKX3.1 expression levels) were shown in enhanced gain to assist in visualization of the red signal. **E** AURKA inhibition does not alter the subcellular localization of NKX3.1 in C4-2 cells. Immunofluorescence images representing the subcellular distribution of NKX3.1 (red) in response to Alisertib in C4-2 cells. The blue channel represents DAPI for the nuclear counterstain. (Scale bar = 20 μm). Images for DMSO treated cells (having much lower relative NKX3.1 expression levels) were shown in enhanced gain to assist in visualization of the red signal. **F** AURKA depletion does not affect the subcellular localization of NKX3.1 in 22Rv1 cells. Immunofluorescence analysis of 22Rv1 cells with and without AURKA knockdown. Texas Red was used for probing NKX3.1 and DAPI (blue) is used for nuclear counterstain. (Scale bar = 20 μm). Western blot for confirmation of AURKA knockdown in 22Rv1 cells. **G** Inhibition of AURKA activity has no effect on the subcellular localization of NKX3.1 in 22Rv1 cells. Images obtained from immunofluorescence microscopy with red—NKX3.1, blue—DAPI. (Scale bar = 20 μm). The images for DMSO treated cells, that have much lower NKX3.1 expression levels than Alisertib treated cells, were shown in enhanced gain to assist in visualization of the red signal. **H** AURKA does not regulate NKX3.1 subcellular residence in C4-2 cells. Subcellular fractionation of NKX3.1 in C4-2 cells in response to knockdown of AURKA is in agreement with immunofluorescence analysis. **I** AURKA does not regulate NKX3.1 subcellular residence in 22Rv1 cells. **J** NKX3.1 does not regulate AURKA’s subcellular residence in C4-2 cells. Scale bar equals 20 µM. AURKA (green) and nucleus (blue). **K** NKX3.1 does not regulate AURKA’s subcellular residence in 22Rv1 cells. Subcellular localization of AURKA did not change when NKX3.1 was silenced. Scale bar equals 20 µM. **L** Subcellular fractionation of AURKA in C4-2 confirms immunofluorescence analysis. Actin and lamin A were used as controls for cytoplasmic and nuclear fractions, respectively. **M** Subcellular fractionation of AURKA in 22Rv1 cells in response to knockdown of NKX3.1 agrees with immunofluorescence analysis. All experiments were conducted at least three independent times

As NKX3.1 was identified as an AURKA target in a global screen, we inspected whether AURKA directly phosphorylates NKX3.1 in vitro. AURKA in complex with its activator TPX2 was incubated with recombinant 6x-His-NKX3.1, which resulted in the phosphorylation of the latter, indicating that NKX3.1 is a substrate of AURKA (Fig. 1A, lane 3).

### AURKA associates with NKX3.1 in CRPC cells

To assess the relevance of AURKA-mediated phosphorylation of NKX3.1 in vitro, we determined whether these two proteins associate in the cellular milieu. AURKA immune complex isolated from C4-2 cells pulled down NKX3.1 (Fig. 1B, lane 3), whereas IgG antibody negative control showed no association (Fig. 1B, lane 1). Similarly, NKX3.1 antibody brought down AURKA, indicating that NKX3.1 and AURKA bind to each other in cells (Fig. 1C, lane 2).

### NKX3.1’s nuclear residence is independent of AURKA

We examined whether AURKA regulates the subcellular location of NKX3.1. AURKA was knocked-down in C4-2 cells, which did not impact the nuclear residence of NKX3.1. Similarly, AURKA inhibition using Alisertib had no effect on NKX3.1 localization (Fig. 1D, E). Equivalent results were observed in 22Rv1 cells, where AURKA silencing or inhibition showed no change in NKX3.1 localization (Fig. 1F, G). To confirm these results, we performed subcellular fractionation in scrambled shRNA and AURKA-shRNA treated C4-2 and 22Rv1 cells. Neither of the cell-type showed any change in NKX3.1 localization upon AURKA silencing (Fig. 1H, I), thereby confirming that NKX3.1 nuclear residence is not controlled by AURKA in cells.

### AURKA subcellular residence is independent of NKX3.1

Unlike NKX3.1, AURKA was present both in the cytoplasm and nucleus in C4-2 and 22Rv1 cells, although it was predominantly cytoplasmic (Fig. 1J, K). NKX3.1 knockdown had no impact on AURKA localization, indicating that it does not regulate AURKA localization in cells. Subcellular fractionation further confirmed these results in both C4-2 and 22Rv1 cells (Fig. 1L, M).

### AURKA downregulates NKX3.1 protein, but not its transcripts

**Fig. 2:**
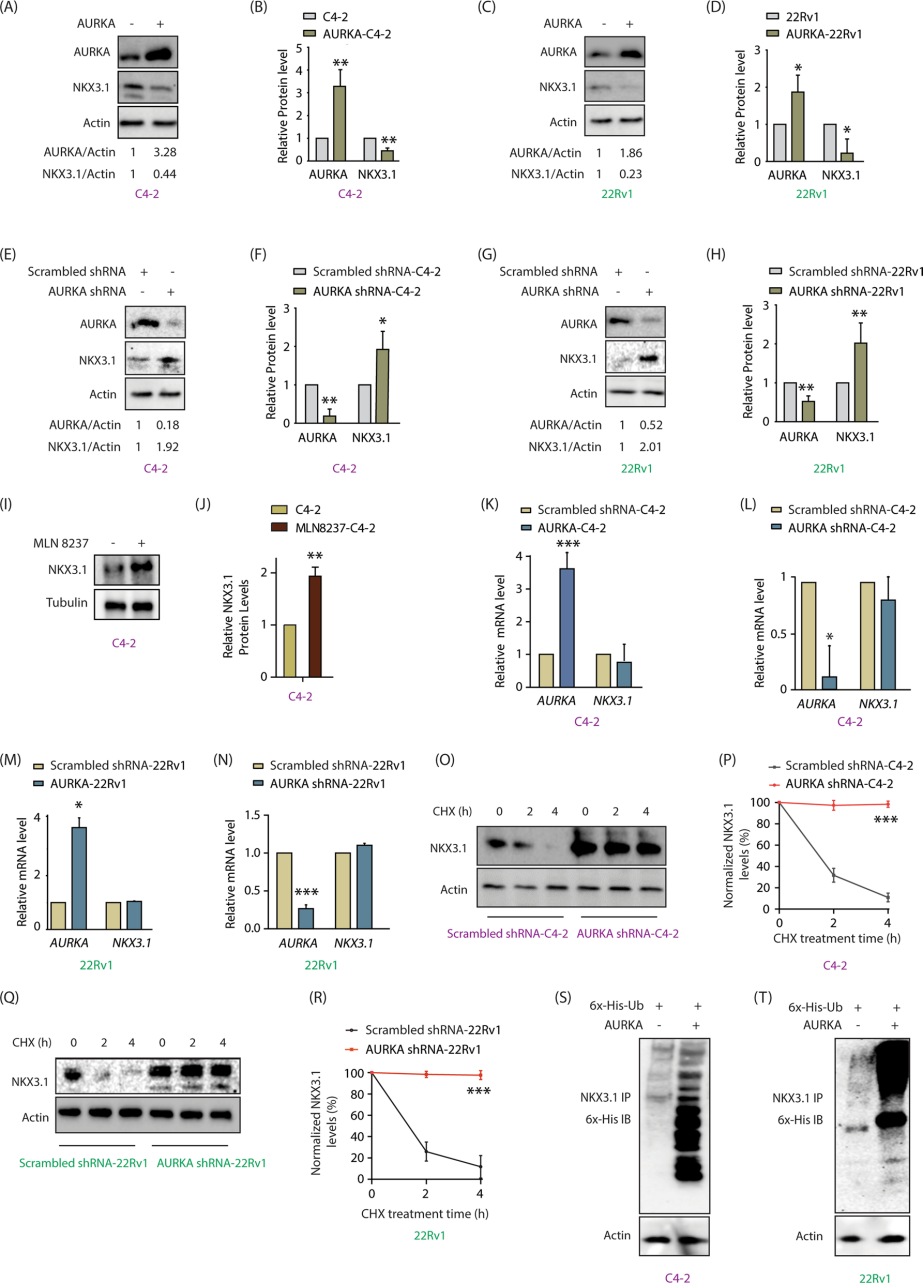
AURKA negatively regulates NKX3.1’s levels by accelerating its ubiquitylation. **A** AURKA negatively regulates NKX3.1 protein level. AURKA was overexpressed in C4-2 cells, and AURKA and NKX3.1 levels analyzed. **B** Quantitative analysis of AURKA, NKX3.1 and actin levels upon AURKA overexpression from three independent experiments. The data were normalized to the actin. **P < 0.01. **C** Overexpression of AURKA decreases NKX3.1 protein level in 22Rv1 cells. **D** AURKA, NKX3.1 and actin levels were analyzed upon AURKA overexpression from three independent experiments in 22Rv1 cells. *P < 0.05. **E** AURKA knockdown increases NKX3.1 protein levels. C4-2 cells were infected with AURKA shRNA lentivirus. **F** Histogram representing the quantitative analysis of detected protein levels from three independent experiments. *P < 0.05, **P < 0.01. **G** Downregulation of AURKA increases NKX3.1 protein level in 22Rv1 cells. **H** The graph shows the statistical analysis of the detected protein from three independent experiments. **P < 0.01. **I** Inhibition of AURKA kinase activity led to elevation of NKX3.1 levels . **J** Quantification of three independent sets of data represents the extent of increase in NKX3.1 protein levels with inhibition of AURKA using MLN8237 (1 µM for 12 h) in C4-2 cells. **P < 0.01 relative to control. **K** AURKA overexpression does not alter NKX3.1 mRNA levels in C4-2 and **L** 22Rv1 cells. The cells were treated with AURKA retrovirus and mRNA levels were analyzed by qRT–PCR from three independent experiments. *P < 0.05, ***P < 0.001. **M** AURKA silencing did not affect NKX3.1 mRNA level in C4-2 cells and **N** 22Rv1 cells. The data were normalized to actin. *P < 0.05, ***P < 0.001. **O** AURKA knockdown stabilizes NKX3.1. C4-2 cells were infected with AURKA shRNA lentivirus following CHX (20 µg/ml) treatment for 2 and 4 h. **P** Dot plot showing NKX3.1 protein levels from three independent experiments using AURKA shRNA and CHX treated cells as indicated in O. ***P < 0.001. **Q** AURKA knockdown stabilizes NKX3.1 in 22Rv1 cells. The cells were treated as indicated in O. **R** The dot plot represents the statistical analysis from three independent experiments of AURKA knockdown in 22Rv1 cells. ***P < 0.001. **S** Ectopic expression of AURKA results in NKX3.1 ubiquitination in C4-2 and (**T**) 22Rv1 cells. Cells were infected with AURKA retrovirus and 6x-His-Ub retrovirus for 24 h, and then treated with MG132 for an additional 12 h. NKX3.1 was isolated, and ubiquitylation was detected using a 6x-His antibody

AURKA is overexpressed and NKX3.1 is downregulated in PCa including in CRPC and NEPC. Therefore, we investigated if these two events are related. AURKA overexpression decreased NKX3.1 protein levels in C4-2 cells (Fig. 2A). Figure 2B shows NKX3.1 and AURKA levels from C4-2 and AURKA-C4-2 cells from three independent experiments. Identical results were obtained in 22Rv1 cells, where AURKA overexpression decreased NKX3.1 (Fig. 2C, D). AURKA silencing increased NKX3.1 levels in both C4-2 and 22Rv1 cells, confirming that AURKA decreases NKX3.1 protein (Fig. 2E–H). To further investigate whether AURKA regulates NKX3.1 using its kinase activity, we inhibited AURKA using Alisertib (aka MLN8237), and measured NKX3.1 levels. AURKA inhibition robustly upregulated NKX3.1 levels, confirming that AURKA kinase activity is involved in NKX3.1 regulation (Fig. 2I, J).

We next examined whether AURKA-mediated regulation of NKX3.1 occurs at the mRNA stage. AURKA overexpression resulted in over threefold increase in its transcripts, however, NKX3.1 mRNA levels remained the same in C4-2 cells (Fig. 2K). AURKA silencing also failed to change NKX3.1 mRNA levels in C4-2 cells (Fig. 2L). 22Rv1 cells showed analogous results upon AURKA overexpression and knock-down, respectively (Fig. 2M, N).

### AURKA destabilizes NKX3.1 by ubiquitylating it

We examined NKX3.1 protein stability in AURKA-silenced C4-2 cells. Treatment with cycloheximide, showed that AURKA silencing significantly increased its stability as compared to control cells (Fig. 2O). Figure 2P shows NKX3.1 levels at 0, 2 and 4 h post-cycloheximide treatment of C4-2 and AURKA shRNA-treated C4-2 cells from three independent experiments. 22Rv1 cells also showed enhanced stability of NKX3.1 upon AURKA knock-down (Fig. 2Q, R). Finally, we investigated whether AURKA degrades NKX3.1 via ubiquitylation. AURKA was overexpressed in C4-2 and 22Rv1 cells, which significantly increased the ubiquitylation of NKX3.1 in both cases (Fig. 2S, T). Together, these results confirm that AURKA downregulates NKX3.1 by promoting its ubiquitylation.

### NKX3.1 downregulates AURKA protein, but not its transcripts

**Fig. 3:**
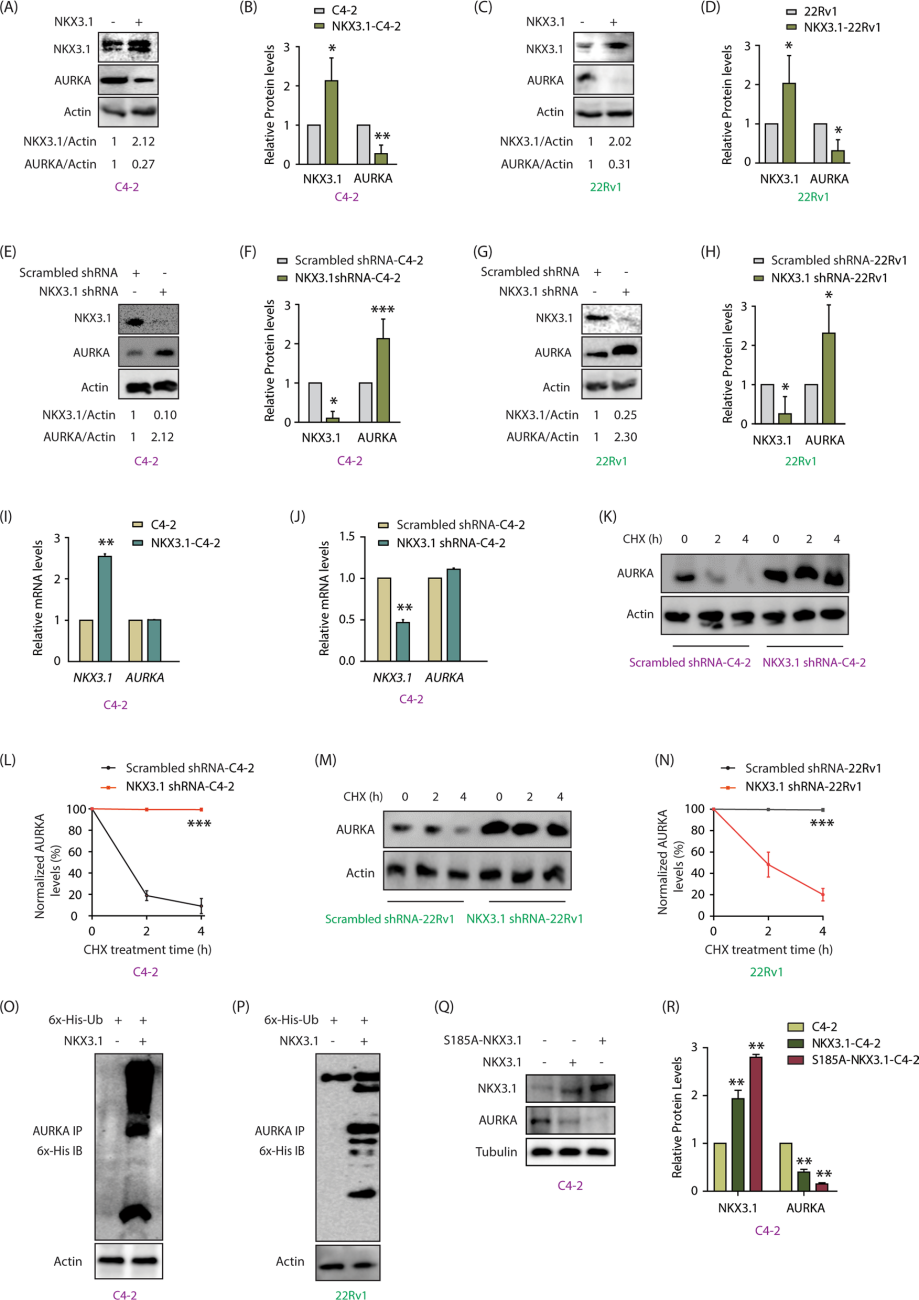
NKX3.1 negatively regulates AURKA’s protein levels by promoting its ubiquitylation. **A** NKX3.1 overexpression decreases AURKA protein levels in C4-2 cells. **B** The graph shows statistical analysis of protein levels from three independent experiments normalized to the actin. *P < 0.05, **P < 0.01. **C** NKX3.1 overexpression decreases AURKA protein levels in 22Rv1 cells. **D** The graph represents the quantitative analysis of protein levels from three independent experiments. *P < 0.05. **E** NKX3.1 silencing increases AURKA level in C4-2 cells. **F** The histogram shows mean ± SD from three independent experiments upon NKX3.1 silencing. *P < 0.05 and ***P < 0.001. **G** NKX3.1 silencing increases AURKA level in 22Rv1 cells. **H** Quantitative analysis of AURKA and NKX3.1 protein levels from three independent experiments. Signals are normalized to the actin. *P < 0.05. **I** NKX3.1 overexpression does not impact AURKA mRNA levels in C4-2 cells, ** P < 0.01. **J** NKX3.1 knockdown does not change AURKA mRNA levels in C4-2 cells, **P < 0.01. **K** Silencing of NKX3.1 stabilizes AURKA protein. C4-2 cells were infected with NKX3.1 shRNA lentivirus for 30 h, followed by CHX (20 µg/ml) treatment for 2 and 4 h. **L** Dot plot showing mean ± SD from three independent experiments upon NKX3.1 silencing. ***P < 0.001. **M** Silencing of NKX3.1 stabilizes AURKA protein in 22Rv1 cells. **N** Dot plot depicting mean ± SD from three independent experiments upon NKX3.1 silencing. ***P < 0.001. **O** NKX3.1 overexpression increases AURKA ubiquitylation in C4-2 and **P** 22Rv1 cells. **Q** Ectopic overexpression of S185A-NKX3.1 curtails AURKA protein levels to a greater extent than WT-NKX3.1 as demonstrated by Western blot analysis. **R** Three independent set of experiments were used for quantification and data plotted as mean ± SEM, **P < 0.01

We have shown that many AURKA substrates regulate it in a feedback manner [9–14], which urged us to inspect whether NKX3.1 controls AURKA. NKX3.1 overexpression indeed robustly decreased AURKA protein in both C4-2 and 22Rv1 cells (Fig. 3A–D). Similarly, NKX3.1 silencing led to a robust increase in AURKA protein levels in both cell types (Fig. 3E–H). In contrast, when NKX3.1 was overexpressed or knocked-down in C4-2 cells, it did not impact the mRNA levels of AURKA, proposing that NKX3.1 does not control AURKA mRNA levels (Fig. 3I, J).

### NKX3.1 destabilizes AURKA

AURKA protein stability was examined in scrambled and NKX3.1 shRNA-expressing C4-2 cells by inhibiting the protein synthesis using cycloheximide. NKX3.1 silencing led to a hugely stabilized AURKA as compared to control cells (Fig. 3K). Figure 3L shows AURKA levels at 0, 2 and 4 h following cycloheximide treatment in scrambled shRNA-treated and NKX3.1 shRNA-treated C4-2 cells from three independent experiments. 22Rv1 cells also showed analogous enhanced stability of AURKA upon NKX3.1 silencing (Fig. 3M, N). We explored whether NKX3.1 degrades AURKA via ubiquitylation. NKX3.1 overexpression in C4-2 and 22Rv1 cells considerably increased the ubiquitylation of AURKA, thereby confirming that like AURKA-mediated regulation, NKX3.1 also facilitates the ubiquitylation of AURKA (Fig. 3O, P).

To further confirm that NKX3.1 overexpression causes AURKA degradation, we overexpressed S185A-NKX3.1 mutant in C4-2 cells, which is more stable than WT, and thus is expressed at relatively higher levels in cells [28]. While WT NKX3.1 expression decreased AURKA levels, S185A expression decreased it even more significantly, confirming that NKX3.1 downregulates AURKA levels in a dose-dependent manner (Fig. 3Q, R).

### AURKA phosphorylates NKX3.1 at S28, S101 and S209

**Fig. 4:**
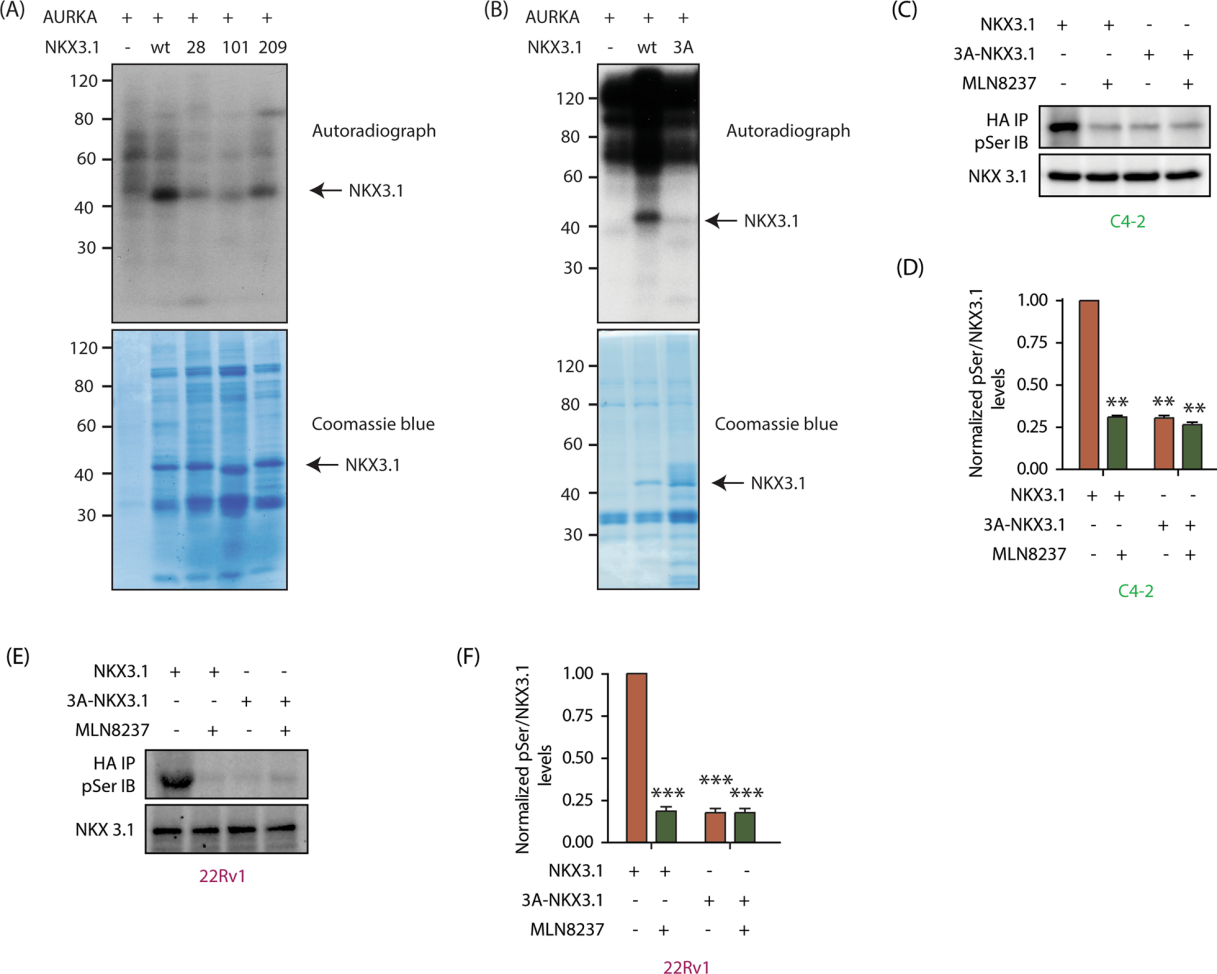
AURKA phosphorylates NKX3.1 via direct phosphorylation at S28, 101, and 209 in vitro and in C4-2 and 22Rv1 cells. **A** AURKA phosphorylates NKX3.1 at S28, S101 and S209. Kinase assays were conducted as indicated in Materials and Methods. **B** S28, S101 and S209 are the only AURKA sites on NKX3.1, as the phospho-resistant triple mutant (3A-NKX3.1) is not phosphorylated by AURKA. The top panel is the autoradiograph. The bottom panel shows the corresponding Coomassie blue-stained gel. **C** AURKA phosphorylates NKX3.1 at S28, S101 and S209 in C4-2 cells. Ectopically expressed HA-tagged NKX3.1 was pulled down using HA antibody and phospho-serine levels of NKX3.1 were measured in NKX3.1-C4-2 along with 3A-NKX3.1-C4-2 cells in response to inhibition of AURKA (1 μM MLN8237, 12 h). **D** Quantification of p-Ser levels (relative to total NKX3.1 levels) obtained from three independent experiments. All values were normalized against wild-type NKX3.1 overexpressing cells without AURKA inhibition (**P < 0.01). **E** S28, S101 and S209 are the only three sites of AURKA-mediated phosphorylation of NKX3.1 in 22Rv1 cells. NKX3.1-22Rv1 and 3A-NKX3.1-22Rv1 cells were either treated with DMSO control or 1 μM AURKA inhibitor (MLN8237) for 12 h, followed by NKX3.1 immunoprecipitation using HA antibody after which phospho-Ser and NKX3.1 levels were probed using Western blot analysis. **F** Quantification of pSer levels of NKX3.1 in 22Rv1 cells in response to AURKA inhibition. The bar graph is representative of data obtained from three independent experiments, normalized to NKX3.1-22Rv1 cells without AURKA inhibition, (***P < 0.001)

Based on the AURKA consensus site determined using peptide substrates [36], we hypothesized S28, S101 and S209 as putative phosphorylation sites on NKX3.1. Therefore, we generated the corresponding 6x-His-tagged phospho-resistant single mutants (S28A-NKX3.1, S101A-NKX3.1 and S209A-NKX3) and exposed them to an in vitro kinase assay using recombinant AURKA-TPX2 complex. Wild-type (WT) NKX3.1 was used as a positive control. As shown, all three phospho-dead mutants displayed reduced phosphorylation as compared to WT-NKX3.1, indicating that AURKA phosphorylates all three sites in vitro (Fig. 4A).

To confirm that S28, S101 and S209 are the only sites that are phosphorylated by AURKA, we generated the respective triple mutant (3A-NKX3.1) and performed an in vitro kinase assay using AURKA-TPX2. While WT NKX3.1 was robustly phosphorylated, 3A-NKX3.1 showed minimal phosphorylation, confirming that AURKA only phosphorylates these three sites on NKX3.1 (Fig. 4B).

We subsequently tested whether AURKA phosphorylates NKX3.1 in cells. HA-tagged WT and 3A-NKX3.1 were ectopically expressed in C4-2 cells, followed by alisertib treatment for 12 h. HA-tagged proteins were isolated and their phospho-Ser levels were analyzed using a phospho-Ser antibody. While WT NKX3.1 showed robust phosphorylation, it was completely abolished upon alisertib treatment, indicating that NKX3.1 is phosphorylated by AURKA in C4-2 cells (Fig. 4C). Furthermore, 3A-NKX3.1 showed minimal phosphorylation, which was not affected by alisertib treatment. These results thus show that AURKA phosphorylates NKX3.1 at these sites in C4-2 cells. Figure 4D shows WT and 3A-NKX3.1 phospho-levels from control and alisertib-treated C4-2 cells from three independent experiments. We observed similar phosphorylation of NKX3.1 by AURKA in 22Rv1 cells, indicating that NKX3.1 is a bonafide substrate of AURKA in CRPC cells (Fig. 4E, F).

### Phospho-resistant NKX3.1 shows significantly enhanced stability

**Fig. 5:**
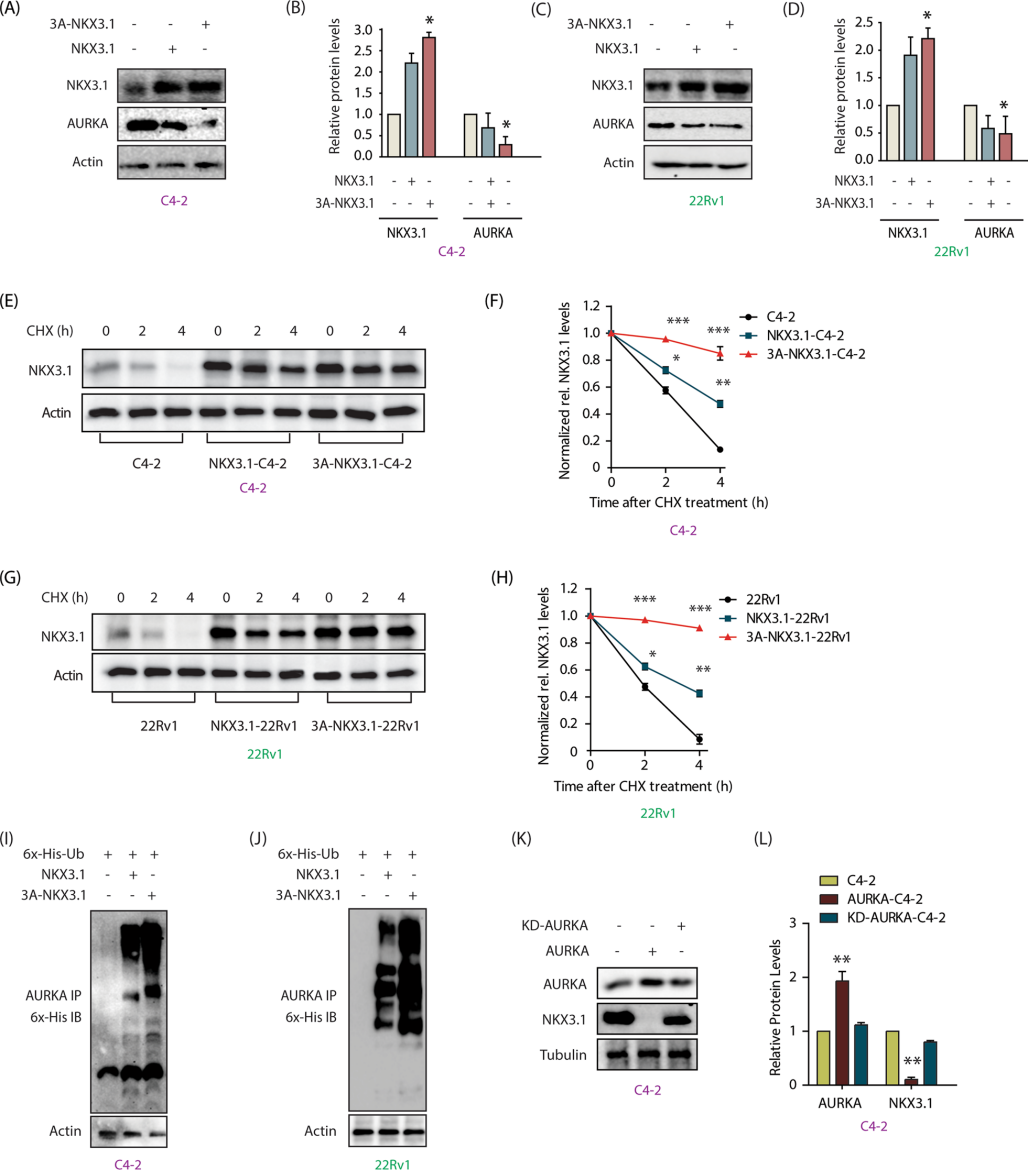
AURKA modulates NKX3.1 stability via direct phosphorylation at S28, 101, and 209. **A** 3A-NKX3.1 shows higher expression as compared to NKX3.1 in C4-2 cells. **B** Quantification of NKX3.1 levels normalized to actin from three independent experiments. *P < 0.05. **C** 3A-NKX3.1 displays higher expression as compared to wild-type in 22Rv1 cells. **D** Quantification of NKX3.1 levels normalized to actin from three independent experiments in 22Rv1 cells. *P < 0.05. **E** CHX experiments revealed higher stability of 3A-NKX3.1 as compared to WT NKX3.1 in C4-2 cells. **F** Graphical representation showing mean ± SD from three independent experiments. *P < 0.05, **P < 0.01, ***P < 0.001. **G** CHX experiments revealed higher stability of 3A-NKX3.1 as compared to WT NKX3.1 in 22Rv1 cells. **H** Quantification shows mean ± SD from three independent experiments. *P < 0.05, **P < 0.01, ***P < 0.001. **I** 3A-NKX3.1 most efficiently ubiquitylates AURKA as compared to control C4-2 and WT NKX3.1-C4-2 cells. **J** 3A-NKX3.1 most efficiently ubiquitylates AURKA as compared to control 22Rv1 and WT NKX3.1-22Rv1 cells. **K** Ectopic overexpression of kinase-dead AURKA does not alter NKX3.1 protein levels substantially . **L** Quantitative analysis of three independent experiments using WT and kinase-dead (KD) AURKA reflect the protein levels of NKX3.1 in C4-2 cells, **P < 0.01 relative to control

We next tested the consequences of AURKA-triggered phosphorylation of NKX3.1. Both WT and 3A-NKX3.1 were ectopically expressed in C4-2 cells. As expected, phospho-resistant 3A-NKX3.1 showed higher levels as compared to WT (Fig. 5A, B). Conversely, AURKA levels showed the opposite pattern with the highest levels in control C4-2 cells, followed by WT and least in 3A-NKX3.1 cells, confirming the negative regulation by NKX3.1. We observed a similar pattern in 22Rv1 cells (Fig. 5C, D).

The stability of WT and 3A mutant was compared using cycloheximide. As shown, 3A-NKX3.1 showed a substantially longer half-life as compared to the WT allele (Fig. 5E). Figure 5F shows WT and 3A-NKX3.1 degradation patterns from three independent experiments. Comparable regulation was observed in 22Rv1 cells (Fig. 5G, H).

As enhanced NKX3.1 levels are expected to degrade AURKA, we analyzed relative ubiquitylation of AURKA in control, WT and 3A-NKX3.1 expressing C4-2 and 22Rv1 cells. While WT induced robust ubiquitylation of AURKA, 3A-NKX3.1 triggered even higher ubiquitylation of AURKA, thereby confirming the negative feedback loop between NKX3.1 and AURKA (Fig. 5I, J).

 Our findings showing that AURKA inhibition increases NKX3.1 levels (Fig. 2I, J) and phospho-resistant 3A-NKX3.1 is more stable than WT, strongly indicated that AURKA stabilizes NKX3.1 using its kinase activity. To further confirm this hypothesis, we expressed WT and kinase-inactive (D274N)AURKA, and analyzed NKX3.1 levels. While NKX3.1 levels decreased drastically upon WT AURKA expression, kinase-inactive AURKA had minimal impact on NKX3.1 levels, confirming that AURKA-mediated phosphorylation is responsible for NKX3.1 degradation (Fig. 5K, L).

### Phospho-resistant NKX3.1 inhibits AURKA-induced oncogenic phenotypes

**Fig. 6:**
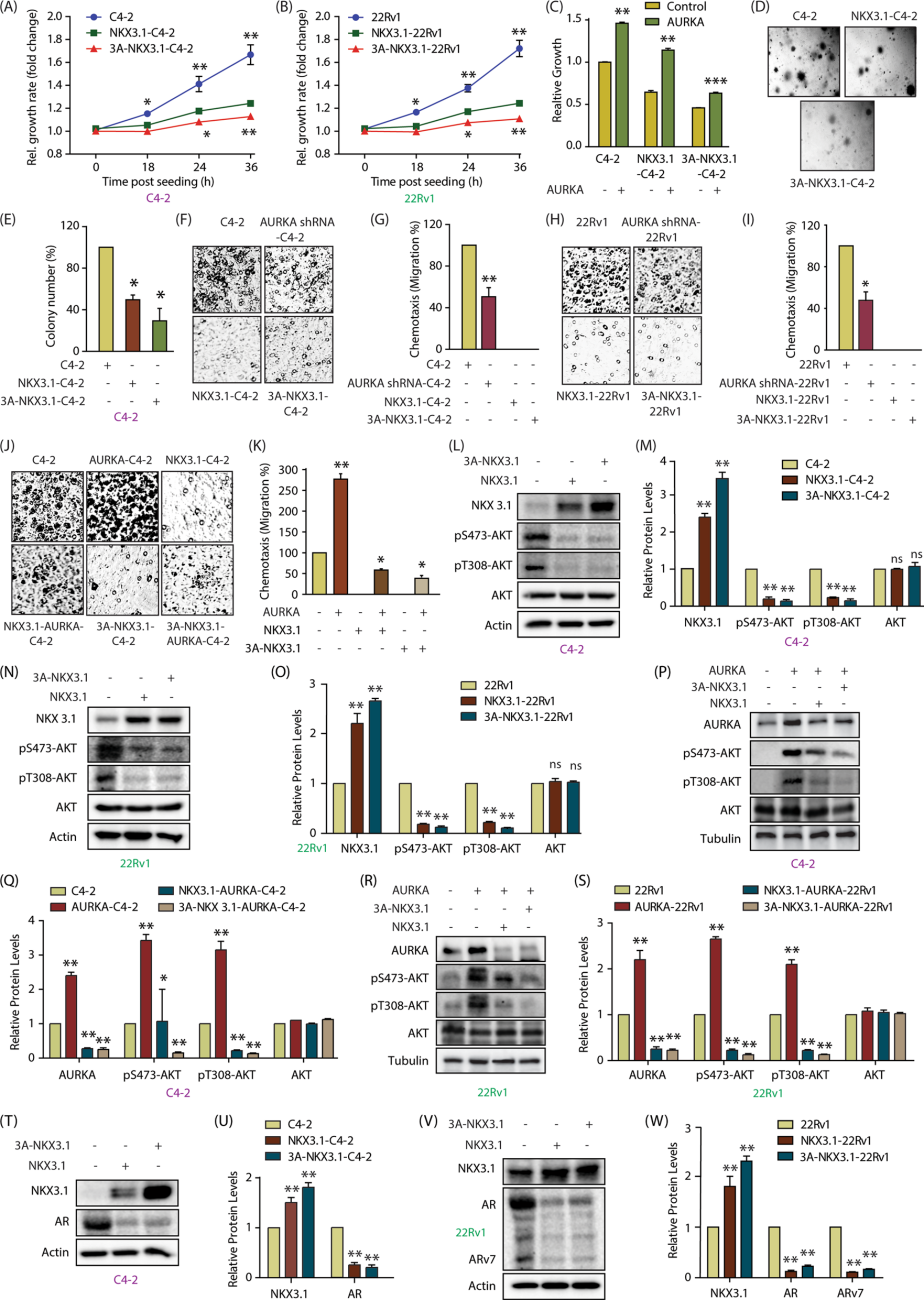
NKX3.1 and AURKA cross-talk regulates aggressive phenotypes including AR, ARv7 upregulation and AKT activation in CRPC cells. **A** Phospho-resistant NKX3.1 inhibits cell proliferation more effectively in C4-2 cells as compared to WT NKX3.1. Cell proliferation was measured at indicated times. *P < 0.05, **P < 0.01. **B** Phospho-resistant NKX3.1 inhibits cell proliferation more effectively in 22Rv1 cells as compared to WT NKX3.1.  *P < 0.05, **P < 0.01. **C** Ectopic expression of AURKA increases cell proliferation in C4-2 and NKX3.1-C4-2 cells, but not in 3A-NKX3.1-C4-2 cells. AURKA retrovirus was transiently infected in C4-2, NKX3.1 and 3A-NKX3.1 cells and cell growth was measured after 36h using MTT assay. **P < 0.01, and ***P < 0.001. **D** Colony formation assay showed that 3A-NKX3.1 is more effective in inhibiting colony formation as compared to the WT allele. **E** Quantitative data analysis of the soft agar experiment from three independent experiments. *P < 0.05. **F** NKX3.1 and 3A-NKX3.1 fully suppress chemotaxis in C4-2 cells, whereas AURKA knockdown partially suppressed it. The cells were starved in serum-free media for 12 h. Chemotaxis was performed using Boyden chambers. **G** The plot shows mean ± SEM of cell motility in C4-2, AURKA-knocked down-C4-2, NKX3.1 and 3A-NKX3.1-C4-2 cells from three independent experiments. **P < 0.01. **H** NKX3.1 and 3A-NKX3.1 fully suppress chemotaxis in 22Rv1 cells, whereas AURKA knockdown partially suppressed it. **I** Bar graph indicating the extent of migration plotted as mean ± SD of three independent experiments such as the one indicated in **H**. *P < 0.05. **J** AURKA overexpression rescues chemotaxis more effectively in C4-2 and NKX3.1-C4-2 cells, as compared to 3A-NKX3.1-C4-2 cells. **K** Histogram representing the quantification of migration levels, plotted as mean ± SD of three independent experiments. *P < 0.05, **P < 0.01. **L** Levels of phospho-AKT in NKX3.1 and 3A-NKX3.1 overexpressing C4-2 cells are significantly lower than control cells. Control, NKX3.1-C4-2 and 3A-NKX3.1-C4-2 cells were assayed for p-AKT levels along with AKT and actin. **M** Quantification of change in AKT phosphorylation levels in response to NKX3.1 and 3A-NKX3.1-expression. Data from three independent experiments was normalized against actin, and represented as mean ± SEM [**P < 0.01, *ns* not significant]. **N** Degree of AKT phosphorylation is lowered by ectopic overexpression of wild-type and 3A-NKX3.1 in 22Rv1 cells. **O** Quantification of AKT phosphorylation levels obtained from three independent experiments such as the one depicted in **N**. [**P < 0.01, *ns* not significant]. **P** WT and 3A-NKX3.1 retroviruses were infected in AURKA overexpressing C4-2 cells and p-AKT levels were analyzed along with AKT and tubulin. **Q** Data from three independent experiments as in 6P were used for quantification, *P < 0.05, **P < 0.01 relative to control. **R** AURKA overexpressing 22Rv1 cells were also assessed for p-AKT levels in response to WT and 3A-NKX3.1 overexpression. **S** Three independent experiments as in 6R were used for quantitative analysis, **P < 0.01. **T** Both wild-type NKX3.1 and 3A-NKX3.1 deplete AR protein levels in C4-2 cells. **U** Histogram showing change in AR and NKX3.1 protein levels. Normalized data from three independent experiments, with actin as loading control, was plotted, **P < 0.01 compared to control cells. **V** Ectopic expression of NKX3.1 and 3A-NKX3.1 depletes AR protein levels in 22Rv1 cells. **W** Histogram depicting changes in AR protein levels in 22Rv1, NKX3.1-22Rv1 and 3A-NKX3.1-22Rv1 cells. The data from three independent experiments was plotted as mean ± SEM, **P < 0.01 vs 22Rv1 control cells

We next investigated whether phospho-resistant NKX3.1 can reverse the oncogenic phenotypes induced by AURKA. Initially we compared the cell proliferation rates of C4-2, NKX3.1-C4-2 and 3A-NKX3.1 cells. C4-2 showed the highest growth, followed by WT-expressing cells. 3A-NKX3.1-C4-2 cells showed severely reduced cell growth (Fig. 6A). We observed the same trend in 22Rv1 cells (Fig. 6B). We next investigated whether AURKA overexpression rescues NKX3.1-mediated suppression of cellular proliferation. AURKA was ectopically expressed in C4-2, NKX3.1-C4-2 and 3A-NKX3.1-C4-2 cells, and their relative proliferation rates were compared. AURKA overexpression increased proliferation in C4-2 cells as predicted (Fig. 6C). AURKA expression also reversed the growth inhibitory effect of WT-NKX3.1 presumably by promoting its degradation. AURKA overexpression also slightly increased the growth rate in 3A-NKX3.1-expressing cells, indicating that AURKA promotes multiple oncogenic pathways, which are independent of NKX3.1 (Fig. 6C).

We also investigated the effect of NKX3.1 phosphorylation by AURKA under anchorage-independent conditions. While control C4-2 cells formed large number of colonies, WT NKX3.1 expression reduced it by 50%, and mutant NKX3.1 by ~ 70%, thereby showing that AURKA-mediated phosphorylation and subsequent degradation of NKX3.1 is an important step in AURKA-mediated malignancy (Fig. 6D, E).

The relative cell motility of NKX3.1-expressing cells was next analyzed. AURKA knock-down decreased cell motility by almost 50% of the control cells (Fig. 6F, G). More importantly, both WT and mutant NKX3.1 completely inhibited chemotaxis, underscoring a major tumor-suppressive role of NKX3.1, one of which includes degrading AURKA. Similarly, overexpression of either WT or mutant NKX3.1 fully abrogated chemotaxis in 22Rv1 cells, underscoring a strong tumor-suppressive function of NKX3.1 in CRPC, whereas AURKA knock-down showed a moderate impact (Fig. 6H, I).

AURKA was further overexpressed in control C4-2, WT and 3A-NKX3.1-C4-2 cells to investigate whether it reverses the negative impact of NKX3.1 in cell motility. AURKA overexpression increased chemotaxis in C4-2 cells as predicted. AURKA could rescue cell motility in NKX3.1-C4-2 cells as well, but not in 3A-NKX3.1 cells, indicating that phospho-resistant NKX3.1 is fully capable of counteracting the oncogenicity of AURKA (Fig. 6J, K). Together, these results implicate that the balance between AURKA and NKX3.1 levels is crucial in dictating the aggressiveness of PCa tumors.

### AURKA upregulates AKT, AR and ARv7 signaling via NKX3.1 phosphorylation

Activation of the PI3K-AKT pathway plays a critical role in the initiation and progression of CRPC. As NKX3.1 inhibits the AKT pathway, we investigated whether AURKA activates the AKT pathway via NKX3.1 in CRPC. WT and 3A-NKX3.1 were overexpressed in C4-2 cells, which fully inhibited AKT activation, although there was no change in AKT levels (Fig. 6L, M). We observed similar AURKA-mediated regulation of AKT signaling in 22Rv1 cells (Fig. 6N, O). As both WT and 3A-NKX3.1 fully inhibited phospho-AKT signaling in C4-2 and 22Rv1 cells, we tested the potential impact of these two alleles in AURKA-overexpressing-C42 and 22Rv1 cells. AURKA overexpression strongly increased phospho-AKT signal at S473 and T308 sites in both C4-2 and 22Rv1 cells (Fig. 5P–S), which was robustly decreased by ectopic expression of WT-NKX3.1. 3A-NKX3.1 expression was relatively more effective than WT in diminishing phospho-AKT levels in these cells (Fig. 5P–S).

We next examined whether AURKA also upregulates the AR pathway by degrading NKX3.1. Ectopic expression of WT and mutant NKX3.1 completely abolished AR levels in C4-2 cells (Fig. 6T, U). In 22Rv1 cells, both AR and Arv7 levels were severely reduced upon WT and phospho-resistant NKX3.1 overexpression (Fig. 6V, W). Together, these results revealed a direct link of AURKA in activating AKT and AR pathways in CRPC via degradation of NKX3.1.

### AURKA-NKX3.1 cross-talk in NEPC cells

AURKA amplification is one of the salient features of NEPC, and is causally linked to neuroendocrine differentiation in both de novo and ADT-resistant CRPC tumors [4]. In contrast, NKX3.1 mRNA and protein levels have only been analyzed in a few NEPC tumors, most of which show downregulation of NKX3.1 [37, 38]. These findings prompted us to investigate whether AURKA upregulation could be linked to NKX3.1 levels in NEPC. We also wondered whether NKX3.1 could regulate neuroendocrine phenotypes, which has not been shown in any study to date.

**Fig. 7:**
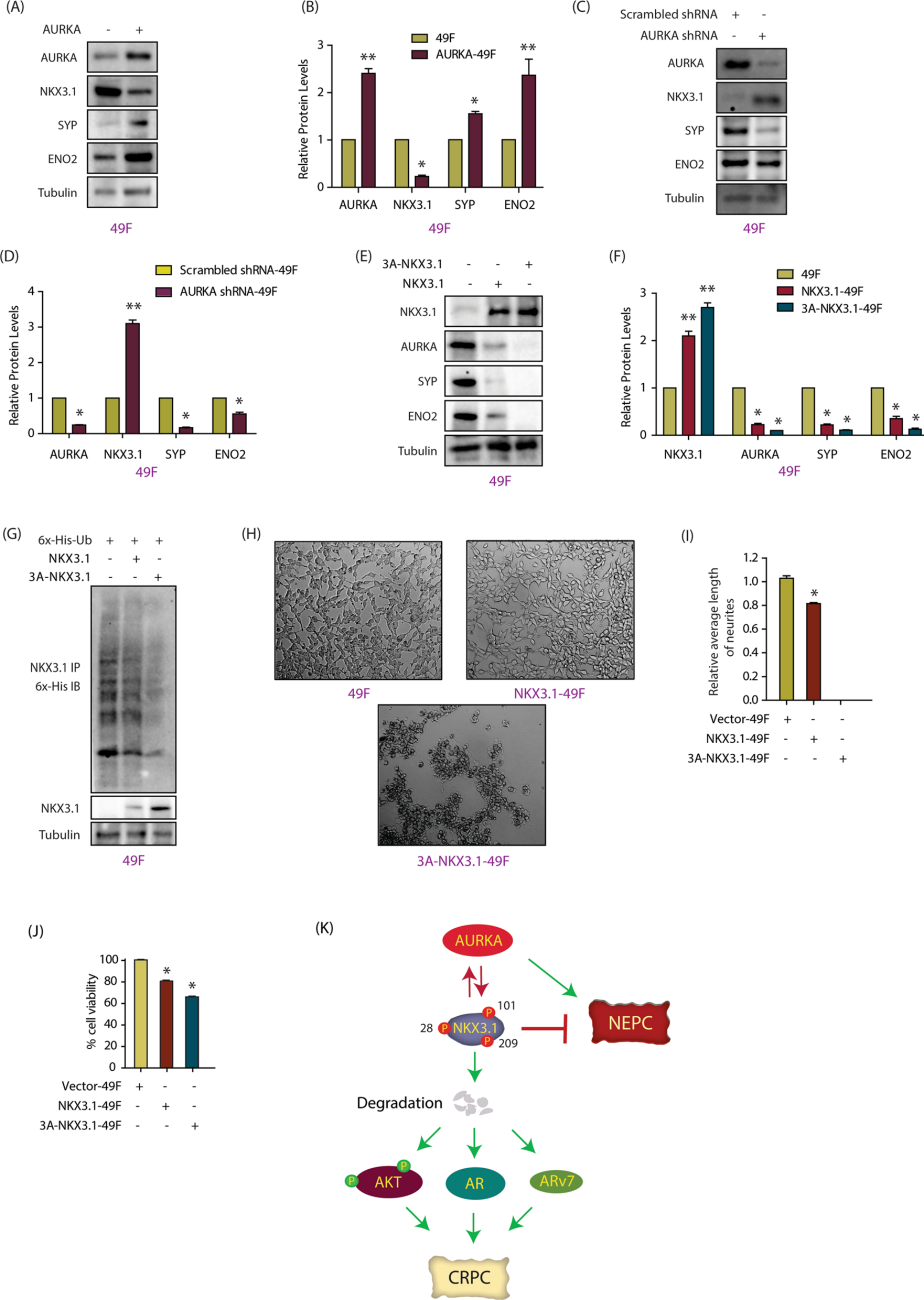
AURKA negatively regulates NKX3.1 in NEPC. **A** AURKA overexpression in 49F cells leads to lowering of NKX3.1 expression levels. **B** Quantitative analysis of AURKA, NKX3.1, synaptophysin, enolase and tubulin levels upon ectopic overexpression of AURKA. The data, obtained from three independent experiments, were normalized to tubulin. *P < 0.05, **P < 0.01. **C** AURKA knockdown enhances NKX3.1 protein levels in 49F cells. **D** Histogram representing the quantitative analysis of detected protein levels from three independent experiments. *P < 0.05, **P < 0.01. **E** 3A-NKX3.1 shows higher expression as compared to NKX3.1 in 49F cells. **F** Quantification of NKX3.1, AURKA, synaptophysin and enolase levels normalized to tubulin from three independent experiments. *P < 0.05, **P < 0.01. **G** 3A-NKX3.1 is most poorly ubiquitylated as compared to control 49F and WT-NKX3.1 overexpressing 49F cells. **H** Micrographs showing changes in neurite outgrowth upon ectopic overexpression of NKX3.1 and 3A-NKX3.1 in 49F cells. **I** Histogram representing neurite length (number of cell body widths). Quantification was performed by observing ten different fields of cells, in five different replicates. **J** Histogram showing effect of WT and 3A-NKX3.1 overexpression on viability of 49F cells. **K** Schematic model describing the plausible pathway of AURKA-NKX3.1 signaling in CRPC and NEPC pathogenesis

We chose 49F cells, which are enzalutamide-resistant AR-positive NEPC cells [39]. AURKA was overexpressed, which led to NKX3.1 downregulation (Fig. 7A, B). Likewise, AURKA silencing increased NKX3.1 levels (Fig. 7C, D), confirming that AURKA regulates NKX3.1 levels in NEPC cells as well. As a control, we analyzed NEPC markers—synaptophysin and enolase, which increased upon AURKA overexpression as expected (Fig. 7A–D). We also examined if NKX3.1-AURKA feedback loop exists in these cells by ectopically expressing vector, WT or mutant NKX3.1. While WT NKX3.1 severely downregulated AURKA, 3A-NKX3.1 expression obliterated AURKA levels (Fig. 7E, F). We further investigated the relative ubiquitylation of WT and 3A-NKX3.1 in 49F cells using vector-infected cells as control. As shown in Fig. 7G, both endogenous and WT-NKX3.1 showed higher ubiquitylation than 3A-NKX3.1, confirming that AURKA is responsible for its degradation in NEPC cells as well.

Just as importantly, we observed that NKX3.1 overexpression fully abolished synaptophysin and enolase expression, uncovering a strong negative relationship between NKX3.1 and neuroendocrine phenotype (Fig. 7E, F). As NKX3.1 has not been linked with neuronal differentiation as yet, we further investigated whether ectopic expression of WT and 3A-NKX3.1 could impact differentiation of neuronal cells. Parental 49F cells showed robust differentiated phenotype with long neurites, which were substantially reduced upon WT-NKX3.1 expression. 3A-NKX3.1 expression fully obliterated differentiated phenotype (Fig. 7H, I). 3A-NKX3.1 cells also showed round apoptotic phenotype with clustering of cells, indicating that 3A-NKX3.1 expression is highly toxic in these cells. These findings prompted us to investigate the relative cell viability of parental, WT-NKX3.1-49F and 3A-NKX3.1-49F cells. The corresponding retroviruses (vector, WT-NKX3.1 and 3A-NKX3.1) were infected in 49F cells, and their viability measured after 24 h. While WT-NKX3.1 expression reduced cellular viability by 20%, 3A-NKX3.1 expression was more toxic with > 35% loss (Fig. 7J). Together, these findings underscore the significance of AURKA-mediated degradation of NKX3.1 in the progression of NEPC pathogenesis.

## Discussion

NKX3.1 is predominantly expressed in prostate luminal epithelial cells, and promotes cellular differentiation and lineage plasticity [22]. The loss of NKX3.1 is a crucial event in PCa initiation. *NKX3.1* is a haploinsufficient gene, and loss of heterozygosity (LOH) at the *Nkx3.1* locus results in hyperplasia and eventually PIN formation. At the molecular level, loss of a single *Nkx3.1* allele prolongs the proliferative stage of dividing luminal cells, causing hyperplasia [40]. Most importantly, the dosage of *Nkx3.1* controls discrete subsets of genes, thus, loss of one Nkx3.1 allele results in complete loss of some target genes, while other genes require loss of both copies [40].

While the loss of NKX3.1 protein is a hallmark of PCa in clinical specimens and mouse models, the accompanying NKX3.1 mRNA levels show little correlation with its protein levels [21]. Additionally, loss of NKX3.1 protein shows little correlation with loss of its locus or with the failure to identify inactivating mutations [41]. All these findings indicate that post-translational regulation of NKX3.1 plays a critical role in diseased states. NKX3.1 protein stability is indeed shown to be differentially regulated by phosphorylation. DYRK1B directly phosphorylates NKX3.1 at S185, which causes its degradation [42]. In contrast, it has been postulated that PIM1 phosphorylates NKX3.1 at both S185 and S186, which increases its stability [43]. CK2 phosphorylates at T89 and T93, which stabilizes it [44]. Markowski et al. showed that during inflammation, tumor necrosis factor (TNF)-alpha and interleukin-1 beta (IL-β) causes NKX3.1 phosphorylation at S196, which promote NKX3.1 degradation, although the kinase was not identified [45]. During DNA damage, active ATM phosphorylates NKX3.1 at residues T134 and T166, accelerating NKX3.1 degradation [46]. PKC (Protein kinase C) was shown to phosphorylate NKX3.1 at S48, although whether it regulates its protein stability is unknown [47]. In contrast to NKX3.1 regulation by several kinases, there is only one report that showed that NKX3.1 also regulates its kinase in a feedback mechanism [28]. We showed that LIMK2 kinase directly phosphorylates NKX3.1 at S185 and degrades it. NKX3.1 in return degrades LIMK2 as well by increasing its ubiquitylation [28]. The present study exposed that AURKA phosphorylates NKX3.1 at S28, S101 and S209, which triggers its ubiquitylation. Thus, regulating NKX3.1’s protein stability is critical both under normal and diseased conditions. Furthermore, as 3A-NKX3.1 is more resistant to AURKA-mediated ubiquitylation, we believe that unlike N-Myc and FOXM1, AURKA uses its kinase activity to degrade NKX3.1. AURKA is highly expressed in C4-2 and 22Rv1 cells regardless of cell cycle, indicating that AURKA-mediated degradation of NKX3.1 is not cell cycle-dependent, although it may increase during mitosis, when AURKA levels are relatively higher. Our results further show that neither the depletion nor inhibition of AURKA has any impact on nuclear localization of NKX3.1. Nevertheless, as AURKA directly regulates NKX3.1 levels via phosphorylation, AURKA is expected to have significant control the transcriptional output of NKX3.1. Future studies are required to fully address AURKA-mediated transcriptional regulation of NKX3.1.

Like NKX3.1, AURKA mRNA levels also show little correlation with protein levels in PCa, underscoring that both their regulation at the protein level is critical. NKX3.1 binds AURKA and triggers its ubiquitylation. 3A-NKX3.1 is resistant to AURKA-mediated degradation, hence, it effectively degrades AURKA and reverses oncogenic phenotypes. Thus, genomic loss of NKX3.1 could be a dominant factor contributing to AURKA protein upregulation in a significant percentage of PCa. While the exact mechanism by which NKX3.1 promotes the ubiquitylation of AURKA remains unclear, it could be at least partly mediated by AKT/GSK3b/FBXW7 pathway [48]. FBXW7 is a F-box protein, which is a part of the substrate recognition component of the SCF E3 ubiquitin ligase. FBXW7 regulates proteasome-mediated degradation of many oncoproteins including AURKA. GSK3β inhibition reduces the binding affinity between AURKA and FBXW7, leading to AURKA stabilization. As NKX3.1 inhibits AKT signaling and AKT inactivates GSK3b by phosphorylation at Ser9, we speculate that NKX3.1 activates GSK3b by inhibiting AKT signaling, leading to AURKA degradation via FBXW7. Future studies are required to validate the exact mechanisms of AURKA degradation by NKX3.1.

Recent studies have uncovered a few mechanisms by which AURKA upregulates AR and ARv7 signaling in CRPC. AURKA stabilizes YBX1 causing upregulation of AR protein and ARv7 mRNA levels [7]. AURKA also degrades SPOP stabilizing both AR and ARv7 proteins [14]. We show that AURKA-mediated NKX3.1 degradation is another mechanism by which AURKA increases both AR and ARv7 levels (Fig. 7K).

Several studies have shown that AURKA inhibition downregulates AKT activation, however, the molecular players mediating this response largely remain unknown. Previously, we identified that AURKA directly phosphorylates and degrades PHLDA1 in breast cancer cells [5]. PHLDA1 is a repressor of AKT signaling, which could lead to AKT activation by AURKA. However, future studies are needed to establish whether PHLDA1 is regulated by AURKA in PCa. This study uncovered that NKX3.1 degradation is a key mechanism by which AURKA augments AKT signaling in CRPC cells (Fig. 7K).

AURKA overexpression or amplification is a hallmark of NEPC. In contrast, little is known about the role of NKX3.1 in NEPC. Nkx3.1 was genomically lost in p53- and Rb-deficient mouse prostate tumors exhibiting neuroendocrine phenotypes [37]. Similarly, human prostate tumors immunoreactive for neuroendocrine markers lacked or minimally showed NKX3.1 immunoreactivity [38]. Although a recent study showed that majority of AR-positive neuroendocrine tumors also express NKX3.1, which is consistent with its origin as an AR-regulated gene [49]. The mechanism by which NKX3.1 could be downregulated at the protein level in NEPC has not been investigated. Our study showed that AURKA overexpression is a major mechanism by which NKX3.1 is downregulated. This is also the first study to show a direct link between NKX3.1 and neuroendocrine phenotype.

## Conclusion

Our studies indicate that NKX3.1 loss could be a major mechanism causing AURKA upregulation in CRPC and NEPC and vice versa. As upregulating NKX3.1 due to genomic loss requires gene therapy, targeting AURKA using specific inhibitors such as alisertib, provides a powerful therapeutic opportunity to maintain NKX3.1 protein levels in CRPC and NEPC. Conversely, when NKX3.1 upregulation strategy using small molecules comes to fruition, AURKA inhibition is expected to work synergistically with NKX3.1 upregulation due to the feedback loop in these highly aggressive diseases.

## Supplementary Information

**Additional file 1. Additional Table S1**. List of antibodies used in this study.** Additional Table S2**. Primer sequences of real-time qPCR primers.

## Data Availability

There are no datasets involved in this study.

## Abbreviations

AURKA: Aurora kinase A
CRPC: Castration-resistant prostate cancer
ADT: Androgen-deprivation therapy
AR: Androgen receptor
CR: Castration-resistant
