# Correction: Reciprocal deregulation of NKX3.1 and AURKA axis in castration-resistant prostate cancer and NEPC models

**DOI:** 10.1186/s12929-025-01189-9

**Published:** 2025-11-10

**Authors:** Moloud Aflaki Sooreshjani, Mohini Kamra, Amina Zoubeidi, Kavita Shah

**Affiliations:** 1 Department of Chemistry and Purdue University Center for Cancer Research, 560 Oval Drive, West Lafayette, IN 47907 USA; 2 Urologic Sciences, University of British Columbia, Vancouver, V6H 3Z6 Canada

**Correction: J Biomed Sci (2021) 28:68** 10.1186/s12929-021-00765-z

After the publication of our article [1], the authors identified errors in Fig. 3G and Fig. 6 (N, O) during figure processing, both the incorrect figures and correct figures are shown below.

The original paper has been updated.

The incorrect Fig. 3:

**Fig. 3:**
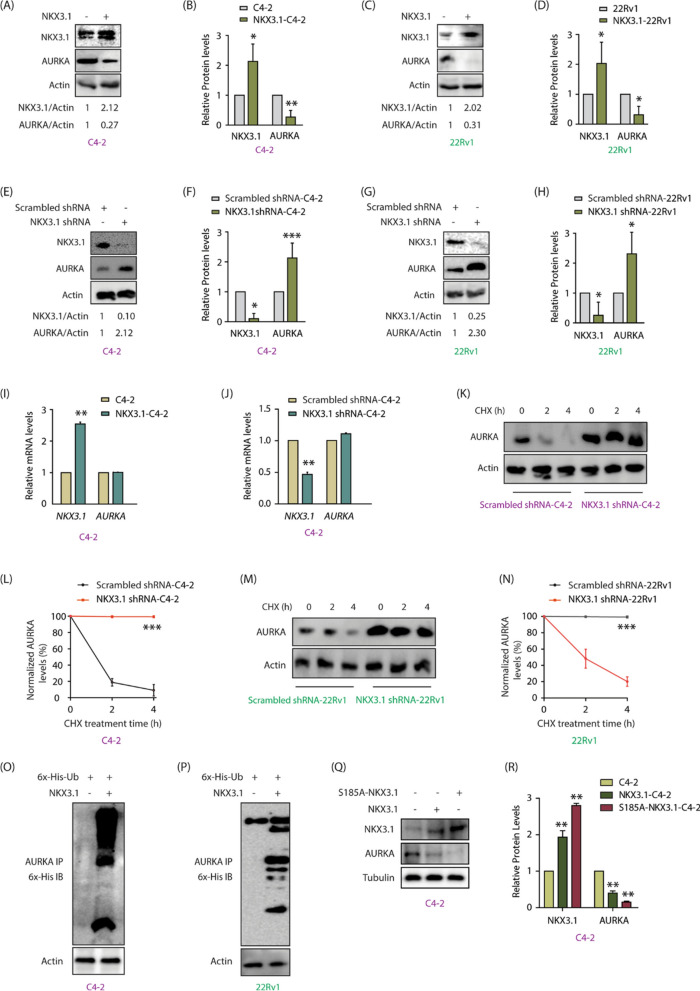
NKX3.1 negatively regulates AURKA’s protein levels by promoting its ubiquitylation. **A** NKX3.1 overexpression decreases AURKA protein levels in C4-2 cells. **B** The graph shows statistical analysis of protein levels from three independent experiments normalized to the actin. *P < 0.05, **P < 0.01. **C** NKX3.1 overexpression decreases AURKA protein levels in 22Rv1 cells. **D** The graph represents the quantitative analysis of protein levels from three independent experiments. *P < 0.05. **E** NKX3.1 silencing increases AURKA level in C4-2 cells. **F** The histogram shows mean ± SD from three independent experiments upon NKX3.1 silencing. *P < 0.05 and ***P < 0.001. **G** NKX3.1 silencing increases AURKA level in 22Rv1 cells. **H** Quantitative analysis of AURKA and NKX3.1 protein levels from three independent experiments. Signals are normalized to the actin. *P < 0.05. **I** NKX3.1 overexpression does not impact AURKA mRNA levels in C4-2 cells, ** P < 0.01. **J** NKX3.1 knockdown does not change AURKA mRNA levels in C4-2 cells, **P < 0.01. **K** Silencing of NKX3.1 stabilizes AURKA protein. C4-2 cells were infected with NKX3.1 shRNA lentivirus for 30 h, followed by CHX (20 μg/ml) treatment for 2 and 4 h. **L** Dot plot showing mean ± SD from three independent experiments upon NKX3.1 silencing. ***P < 0.001. **M** Silencing of NKX3.1 stabilizes AURKA protein in 22Rv1 cells. **N** Dot plot depicting mean ± SD from three independent experiments upon NKX3.1 silencing. ***P < 0.001. **O** NKX3.1 overexpression increases AURKA ubiquitylation in C4-2 and **P** 22Rv1 cells. **Q** Ectopic overexpression of S185A-NKX3.1 curtails AURKA protein levels to a greater extent than WT-NKX3.1 as demonstrated by Western blot analysis. **R** Three independent set of experiments were used for quantification and data plotted as mean ± SEM, **P < 0.01

The correct Fig. 3:

**Fig. 3:**
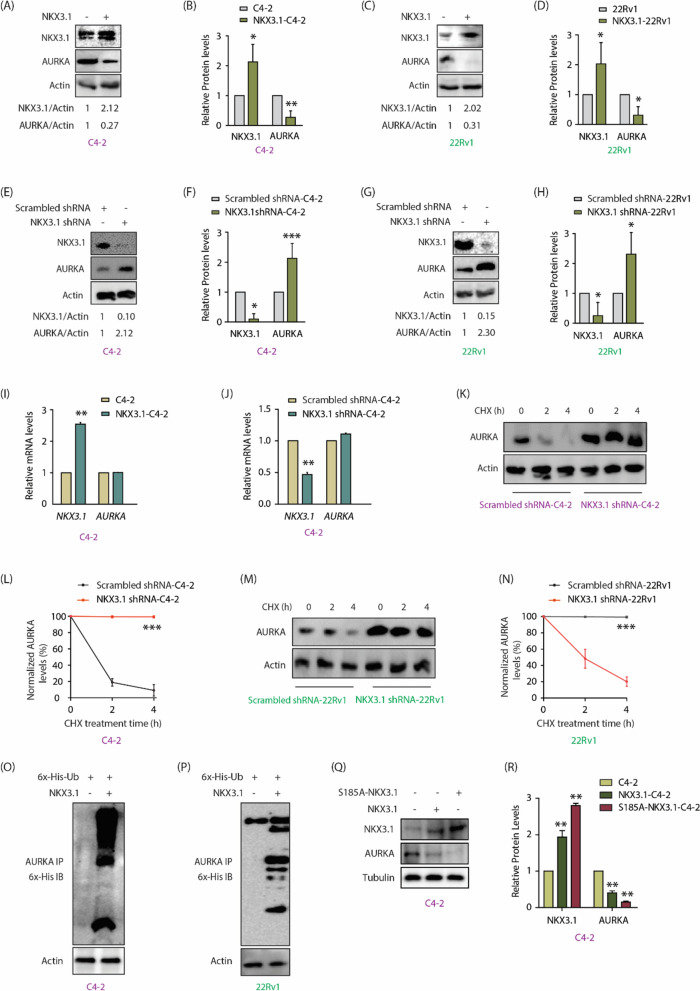
NKX3.1 negatively regulates AURKA’s protein levels by promoting its ubiquitylation. **A** NKX3.1 overexpression decreases AURKA protein levels in C4-2 cells. **B** The graph shows statistical analysis of protein levels from three independent experiments normalized to the actin. *P < 0.05, **P < 0.01. **C** NKX3.1 overexpression decreases AURKA protein levels in 22Rv1 cells. **D** The graph represents the quantitative analysis of protein levels from three independent experiments. *P < 0.05. **E** NKX3.1 silencing increases AURKA level in C4-2 cells. **F** The histogram shows mean ± SD from three independent experiments upon NKX3.1 silencing. *P < 0.05 and ***P < 0.001. **G** NKX3.1 silencing increases AURKA level in 22Rv1 cells. **H** Quantitative analysis of AURKA and NKX3.1 protein levels from three independent experiments. Signals are normalized to the actin. *P < 0.05. **I** NKX3.1 overexpression does not impact AURKA mRNA levels in C4-2 cells, ** P < 0.01. **J** NKX3.1 knockdown does not change AURKA mRNA levels in C4-2 cells, **P < 0.01. **K** Silencing of NKX3.1 stabilizes AURKA protein. C4-2 cells were infected with NKX3.1 shRNA lentivirus for 30 h, followed by CHX (20 μg/ml) treatment for 2 and 4 h. **L** Dot plot showing mean ± SD from three independent experiments upon NKX3.1 silencing. ***P < 0.001. **M** Silencing of NKX3.1 stabilizes AURKA protein in 22Rv1 cells. **N** Dot plot depicting mean ± SD from three independent experiments upon NKX3.1 silencing. ***P < 0.001. **O** NKX3.1 overexpression increases AURKA ubiquitylation in C4-2 and **P** 22Rv1 cells. **Q** Ectopic overexpression of S185A-NKX3.1 curtails AURKA protein levels to a greater extent than WT-NKX3.1 as demonstrated by Western blot analysis. **R** Three independent set of experiments were used for quantification and data plotted as mean ± SEM, **P < 0.01

The incorrect Fig. 6:

**Fig. 6:**
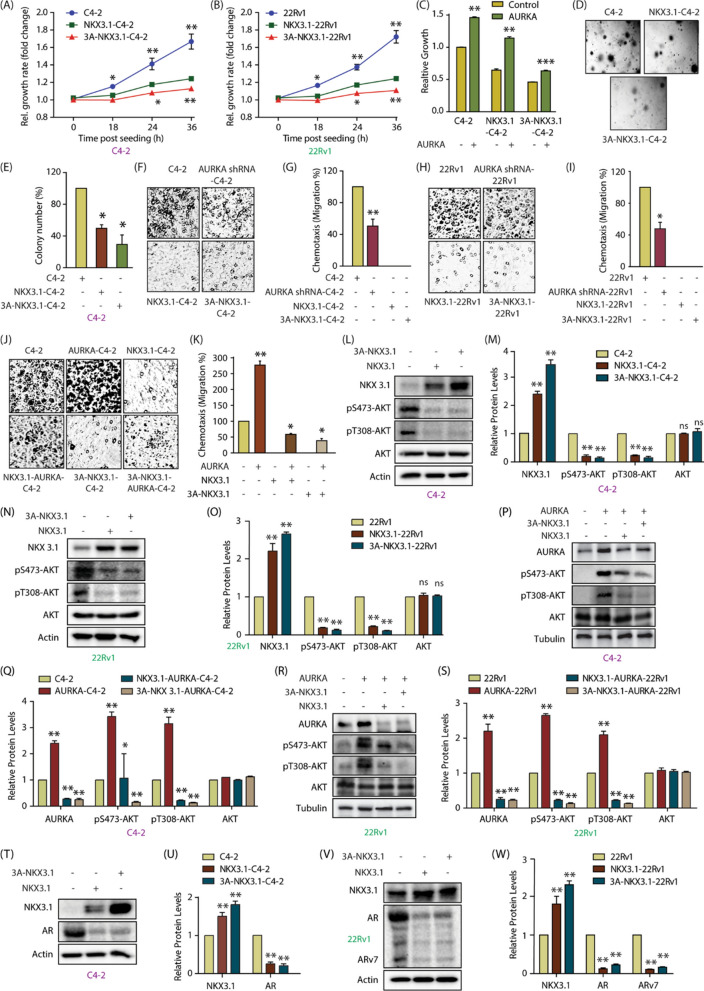
NKX3.1 and AURKA cross-talk regulates aggressive phenotypes including AR, ARv7 upregulation and AKT activation in CRPC cells. **A** Phospho-resistant NKX3.1 inhibits cell proliferation more effectively in C4-2 cells as compared to WT NKX3.1. Cell proliferation was measured at indicated times. *P < 0.05, **P < 0.01. **B** Phospho-resistant NKX3.1 inhibits cell proliferation more effectively in 22Rv1 cells as compared to WT NKX3.1. *P < 0.05, **P < 0.01. **C** Ectopic expression of AURKA increases cell proliferation in C4-2 and NKX3.1-C4-2 cells, but not in 3A-NKX3.1-C4-2 cells. AURKA retrovirus was transiently infected in C4-2, NKX3.1 and 3A-NKX3.1 cells and cell growth was measured after 36h using MTT assay. **P < 0.01, and ***P < 0.001. **D** Colony formation assay showed that 3A-NKX3.1 is more effective in inhibiting colony formation as compared to the WT allele. **E** Quantitative data analysis of the soft agar experiment from three independent experiments. *P < 0.05. **F** NKX3.1 and 3A-NKX3.1 fully suppress chemotaxis in C4-2 cells, whereas AURKA knockdown partially suppressed it. The cells were starved in serum-free media for 12 h. Chemotaxis was performed using Boyden chambers. **G** The plot shows mean ± SEM of cell motility in C4-2, AURKA-knocked down-C4-2, NKX3.1 and 3A-NKX3.1-C4-2 cells from three independent experiments. **P < 0.01. **H** NKX3.1 and 3A-NKX3.1 fully suppress chemotaxis in 22Rv1 cells, whereas AURKA knockdown partially suppressed it. **I** Bar graph indicating the extent of migration plotted as mean ± SD of three independent experiments such as the one indicated in **H**. *P < 0.05. **J** AURKA overexpression rescues chemotaxis more effectively in C4-2 and NKX3.1-C4-2 cells, as compared to 3A-NKX3.1-C4-2 cells. **K** Histogram representing the quantification of migration levels, plotted as mean ± SD of three independent experiments. *P < 0.05, **P < 0.01. **L** Levels of phospho-AKT in NKX3.1 and 3A-NKX3.1 overexpressing C4-2 cells are significantly lower than control cells. Control, NKX3.1-C4-2 and 3A-NKX3.1-C4-2 cells were assayed for p-AKT levels along with AKT and actin. **M** Quantification of change in AKT phosphorylation levels in response to NKX3.1 and 3A-NKX3.1-expression. Data from three independent experiments was normalized against actin, and represented as mean ± SEM [**P < 0.01, *ns *not significant]. **N** Degree of AKT phosphorylation is lowered by ectopic overexpression of wild-type and 3A-NKX3.1 in 22Rv1 cells. **O** Quantification of AKT phosphorylation levels obtained from three independent experiments such as the one depicted in **N**. [**P < 0.01, *ns *not significant]. P WT and 3A-NKX3.1 retroviruses were infected in AURKA overexpressing C4-2 cells and p-AKT levels were analyzed along with AKT and tubulin. **Q** Data from three independent experiments as in 6P were used for quantification, *P < 0.05, **P < 0.01 relative to control. **R** AURKA overexpressing 22Rv1 cells were also assessed for p-AKT levels in response to WT and 3A-NKX3.1 overexpression. **S** Three independent experiments as in 6R were used for quantitative analysis, **P < 0.01. **T** Both wild-type NKX3.1 and 3A-NKX3.1 deplete AR protein levels in C4-2 cells. **U** Histogram showing change in AR and NKX3.1 protein levels. Normalized data from three independent experiments, with actin as loading control, was plotted, **P < 0.01 compared to control cells. **V** Ectopic expression of NKX3.1 and 3A-NKX3.1 depletes AR protein levels in 22Rv1 cells. **W** Histogram depicting changes in AR protein levels in 22Rv1, NKX3.1-22Rv1 and 3A-NKX3.1-22Rv1 cells. The data from three independent experiments was plotted as mean ± SEM, **P < 0.01 vs 22Rv1 control cells

The correct Fig. 6:

**Fig. 6:**
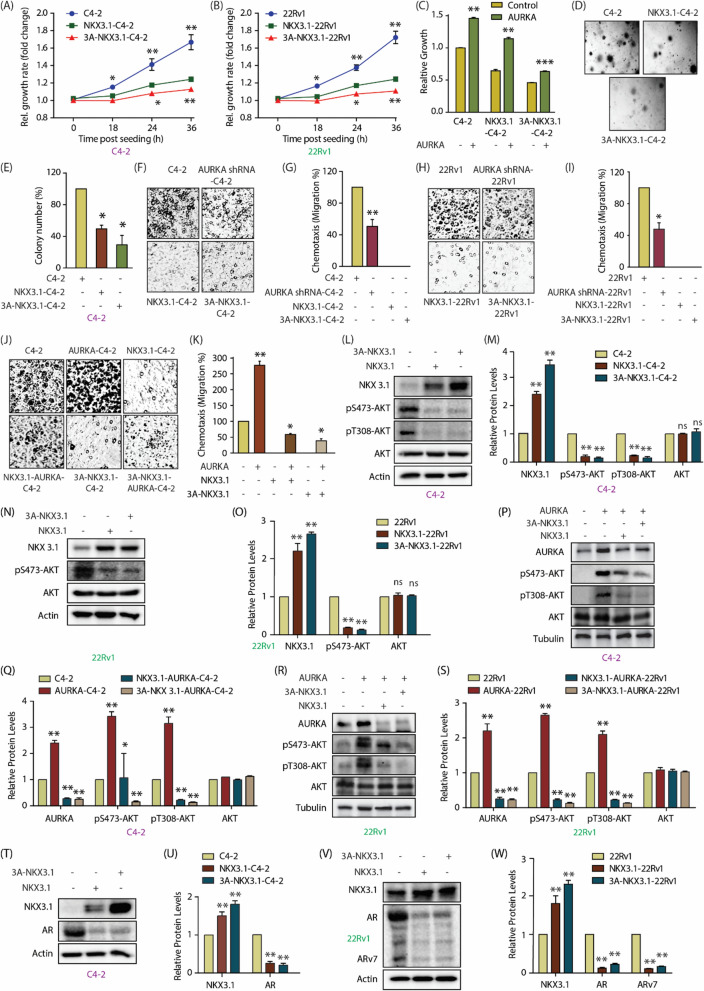
NKX3.1 and AURKA cross-talk regulates aggressive phenotypes including AR, ARv7 upregulation and AKT activation in CRPC cells. **A** Phospho-resistant NKX3.1 inhibits cell proliferation more effectively in C4-2 cells as compared to WT NKX3.1. Cell proliferation was measured at indicated times. *P < 0.05, **P < 0.01. **B** Phospho-resistant NKX3.1 inhibits cell proliferation more effectively in 22Rv1 cells as compared to WT NKX3.1. *P < 0.05, **P < 0.01. **C** Ectopic expression of AURKA increases cell proliferation in C4-2 and NKX3.1-C4-2 cells, but not in 3A-NKX3.1-C4-2 cells. AURKA retrovirus was transiently infected in C4-2, NKX3.1 and 3A-NKX3.1 cells and cell growth was measured after 36h using MTT assay. **P < 0.01, and ***P < 0.001. **D** Colony formation assay showed that 3A-NKX3.1 is more effective in inhibiting colony formation as compared to the WT allele. **E** Quantitative data analysis of the soft agar experiment from three independent experiments. *P < 0.05. **F** NKX3.1 and 3A-NKX3.1 fully suppress chemotaxis in C4-2 cells, whereas AURKA knockdown partially suppressed it. The cells were starved in serum-free media for 12 h. Chemotaxis was performed using Boyden chambers. **G** The plot shows mean ± SEM of cell motility in C4-2, AURKA-knocked down-C4-2, NKX3.1 and 3A-NKX3.1-C4-2 cells from three independent experiments. **P < 0.01. **H** NKX3.1 and 3A-NKX3.1 fully suppress chemotaxis in 22Rv1 cells, whereas AURKA knockdown partially suppressed it. **I** Bar graph indicating the extent of migration plotted as mean ± SD of three independent experiments such as the one indicated in **H**. *P < 0.05. **J** AURKA overexpression rescues chemotaxis more effectively in C4-2 and NKX3.1-C4-2 cells, as compared to 3A-NKX3.1-C4-2 cells. **K** Histogram representing the quantification of migration levels, plotted as mean ± SD of three independent experiments. *P < 0.05, **P < 0.01. **L** Levels of phospho-AKT in NKX3.1 and 3A-NKX3.1 overexpressing C4-2 cells are significantly lower than control cells. Control, NKX3.1-C4-2 and 3A-NKX3.1-C4-2 cells were assayed for p-AKT levels along with AKT and actin. **M** Quantification of change in AKT phosphorylation levels in response to NKX3.1 and 3A-NKX3.1-expression. Data from three independent experiments was normalized against actin, and represented as mean ± SEM [**P < 0.01, *ns *not significant]. **N** Degree of AKT phosphorylation is lowered by ectopic overexpression of wild-type and 3A-NKX3.1 in 22Rv1 cells. **O** Quantification of AKT phosphorylation levels obtained from three independent experiments such as the one depicted in **N**. [**P < 0.01, *ns *not significant]. P WT and 3A-NKX3.1 retroviruses were infected in AURKA overexpressing C4-2 cells and p-AKT levels were analyzed along with AKT and tubulin. **Q** Data from three independent experiments as in 6P were used for quantification, *P < 0.05, **P < 0.01 relative to control. **R** AURKA overexpressing 22Rv1 cells were also assessed for p-AKT levels in response to WT and 3A-NKX3.1 overexpression. **S** Three independent experiments as in 6R were used for quantitative analysis, **P < 0.01. **T** Both wild-type NKX3.1 and 3A-NKX3.1 deplete AR protein levels in C4-2 cells. **U** Histogram showing change in AR and NKX3.1 protein levels. Normalized data from three independent experiments, with actin as loading control, was plotted, **P < 0.01 compared to control cells. **V** Ectopic expression of NKX3.1 and 3A-NKX3.1 depletes AR protein levels in 22Rv1 cells. **W** Histogram depicting changes in AR protein levels in 22Rv1, NKX3.1-22Rv1 and 3A-NKX3.1-22Rv1 cells. The data from three independent experiments was plotted as mean ± SEM, **P < 0.01 vs 22Rv1 control cells
