# Supplementary material for: Reciprocal deregulation of NKX3.1 and AURKA axis in castration-resistant prostate cancer and NEPC models
Source: J Biomed Sci. 2021 Oct 8;28:68. doi: 10.1186/s12929-021-00765-z (PMC8499580; doi:10.1186/s12929-021-00765-z)
Supplement: Supplementary file 1 — Additional file 1. Additional Table S1. List of antibodies used in this study. Additional Table S2. Primer sequences of real-time qPCR primers. [file 12929_2021_765_MOESM1_ESM.pdf]

Additional Table S1: List of antibodies used in this study.

		Catalogue No.	Company	RRID
1	NKX3.1	SC-393190	Santa Cruz Biotech	
2	Actin	SC-8432	Santa Cruz Biotech	RRID:AB_626630
3	AURKA	SC-398814	Santa Cruz Biotech	
4	HA	12CA5	Thermo Fisher	RRID:AB_1958069
5	6x-His	AB-1711	Columbia Bioscience	Lot# PUR01016008
6	AR	SC-816	Santa Cruz Biotech	RRID:AB_ 1563391
7	ARv7	M00542-3	BosterBio	
8	Lamin-A	SC-20680	Santa Cruz Biotech	RRID:AB_648148
9	NKX3.1	83700S	Cell Signaling Tech	RRID: AB_2800027
10	AKT	9272	Cell Signaling Tech	RRID:AB_329828
11	pS473-AKT	SC-293125	Santa Cruz Biotech	RRID: AB_2847909
12	pT308-AKT	9275S	Cell Signaling Tech	RRID:AB_329828
13.	AURKA	14475	Cell Signaling Tech	RRID: AB_2665504

Additional Table S2: Primer sequences of real-time qPCR primers

Names	Primer Sequences
AURKA-F	5'- CCACCTTCGGCATCCTAATA -3'
AURKA-R	5'- TCCAAGTGGTGCATATTCCA -3'
NKX3.1-F	5'- CCGCTTCCAAAGACCTAGAGGA -3'
NKX3.1-R	5'- ACCGTCGTCCTCGGTCCTTGG -3'
β-Actin-F	5'- CATGTACGTTGCTATCCAGGC -3'
β-Actin-R	5'- CTCCTTAATGTCACGCACGAT -3'
